# LMP2 deficiency causes abnormal metabolism, oxidative stress, neuroinflammation, myelin loss and neurobehavioral dysfunctions

**DOI:** 10.1186/s12967-023-04071-0

**Published:** 2023-03-28

**Authors:** Xingyong Chen, Yanguang Mao, Yueting Guo, Dongyun Xiao, Zejing Lin, Yiyi Huang, Ying Chun Liu, Xu Zhang, Yinzhou Wang

**Affiliations:** 1 Department of Neurology, Fujian Provincial Hospital, Shengli Clinical Medical College of Fujian Medical University, Fuzhou, 350001 China; 2 Fujian Academy of Medical Science, Fuzhou, 350001 China; 3 Fujian Key Laboratory of Medical Analysis, Fuzhou, 350001 China; 4 Key Laboratory of Stem Cell Engineering and Regenerative Medicine, School of Basic Medical Sciences, Fujian Medical University, Fuzhou, 350122 China; 5 Fujian Provincial Key Laboratory of Critical Care Medicine, Fuzhou, 350001 China

**Keywords:** Oxidative stress, Blood–brain barrier, Immunoproteasome, Neuroinflammation, Cognitive impairment

## Abstract

**Background:**

Substantial evidence suggests that immunoproteasome is implicated in the various neurological diseases such as stroke, multiple sclerosis and neurodegenerative diseases. However, whether the immunoproteasome itself deficiency causes brain disease is still unclear. Therefore, the aim of this study was to explore the contribution of the immunoproteasome subunit low molecular weight protein 2 (LMP2) in neurobehavioral functions.

**Methods:**

Male LMP2 gene completed knockout (LMP2-KO) and littermate wild type (WT) Sprague–Dawley (SD) rats aged 12-month-old were used for neurobehavioral testing and detection of proteins expression by western blotting and immunofluorescence. A battery of neurobehavioral test tools including Morris water maze (MWM), open field maze, elevated plus maze were used to evaluate the neurobehavioral changes in rats. Evans blue (EB) assay, Luxol fast blue (LFB) and Dihydroethidium (DHE) staining were applied to explore the blood–brain barrier (BBB) integrity, brain myelin damage and brain intracellular reactive oxygen species (ROS) levels, respectively.

**Results:**

We firstly found that LMP2 gene deletion did not cause significantly difference in rats’ daily feeding activity, growth and development as well as blood routine, but it led to metabolic abnormalities including higher levels of low-density lipoprotein cholesterol, uric acid and blood glucose in the LMP2-KO rats. Compared with the WT rats, LMP2-KO rats displayed obviously cognitive impairment and decreased exploratory activities, increased anxiety-like behavior and without strong effects on gross locomotor abilities. Furthermore, multiple myelin loss, increased BBB leakage, downregulation of tight junction proteins ZO-1, claudin-5 and occluding, and enhanced amyloid-β protein deposition were observed in brain regions of LMP2-KO rats. In addition, LMP2 deficiency significantly enhanced oxidative stress with elevated levels of ROS, caused the reactivation of astrocytes and microglials and markedly upregulated protein expression levels of interleukin (IL)-1 receptor-associated kinase 1 (IRAK1), IL-6 and tumor necrosis factor-α (TNF-α) compared to the WT rats, respectively.

**Conclusion:**

These findings highlight LMP2 gene global deletion causes significant neurobehavioral dysfunctions. All these factors including metabolic abnormalities, multiple myelin loss, elevated levels of ROS, increased BBB leakage and enhanced amyloid-β protein deposition maybe work together and eventually led to chronic oxidative stress and neuroinflammation response in the brain regions of LMP2-KO rats, which contributed to the initial and progress of cognitive impairment.

## Introduction

It is generally accepted that the ubiquitin–proteasome-system (UPS) controls numerous cellular pathways including signal transduction, inflammatory processes, cell differentiation and apoptosis by degradation of misfolded or damaged proteins. Due to three proteolytic active subunits β1, β2 and β5, the proteasome exerts caspase-like, trypsin-like, and chymotrypsin-like activity, respectively. However, in cells exposed to a variety of stimulate factors such as inflammatory cytokines (for example, interferon-γ) and ischemia, these three catalytic β-subunits are replaced by their immunosubunits β1i (LMP2/proteasome subunit beta 9, PSMB9), β2i (multicatalytic endopeptidase complex-like 1, MECL-1) and β5i (LMP7) and then forms new subtype of proteasome called the immunoproteasome [1, 2].

The immunoproteasome is regarded as a special type of proteasome which involves in both immune and non-immune responses [2]. Apart from MHC-I antigen processing, immunoproteasome mediate proinflammatory cytokine production observed in different animal disease models. Bone marrowderived dendritic cells from LMP2/β1i-deficient mice infected with influenza virus displayed lower levels of interleukin (IL)-1β, IL-6 and TNF-α compared with the WT counterparts. Our previous study found that LMP2 and LMP7 were evident in astrocytes and microglia/macrophage cells in the ipsilateral ischemic hemisphere after cerebral ischemia/reperfusion in rats, respectively. Furthermore, inhibition of LMP2 by shRNA showed the suppression of neuroinflammation response and reduced infarction volume [3].Similarly, Administration of a high selective proteasome inhibitor PR-957 or LMP7 gene knockout significantly attenuates inflammatory cytokines secretion and disease manifestation observed in animal experimental models of autoimmune encephalomyelitis and rheumatoid arthritis [4, 5]. Taken together, these findings suggest a key role for the immunoproteasome participating in innate immune response and regulation of inflammatory cytokines, which possibly involves in compromised NF-κB signaling or NF-κB-independent signal pathways [6, 7].

Beyond the involvement of the immunoproteasome in the immune system, recent studies have begun to unravel the non-immune functions of the immunoproteasome. Due to the rapid induction properties and the enhanced proteolytic activities compared to the standard proteasome, the immunoproteasome prevents accumulation of the oxidatively-damaged and ubiquitylated proteins and maintains protein homeostasis [1, 8]. For example, oxidized damage proteins were accumulated in the liver and brain of LMP7/β5i- and LMP2/β1i-deficient mice [8]. In addition, increased expression of LMP2 has also been observed in brain of Alzheimer’s disease (AD) [9–11], which is a hallmark of amyloid-β protein deposition accompanying with oxidative stress and neuroinflammation response. A mechanism of amyloid-β protein deposition is due to the inability of the damaged UPS to efficiently remove the excess amyloid-β proteins. From this point of view, increased immunoproteasome expression may have a compensatory effect in protecting neurocytes against stress-induced injury and death by clearing toxic amyloid-β protein more efficiently than the standard proteasome. Increased proteasome activities and messenger RNA and protein expression of immunoproteasome was observed in reactive glias in the cortex of the transgenic mouse AD model [11]. However, these data are in contrast with other studies results [9]. In other words, there is still a controversy about the expression and roles of immunoproteasome in AD context. More interestingly, recent research confirmed a relationship between immunoproteasome and emotion in animal experiment. Gorny X, et al. reported that β5i/LMP7 gene deletion mice displayed more anxiety after mild stress and increased cued fear after fear conditioning compared with the WT mice [12]. These findings suggest that the basal proper formation of immunoproteasome in healthy mice seem to be involved in the regulation of anxiety and cued fear levels. Our previous work found that elevated level of plasma LMP2 during the acute phase of ischemic stroke was high risk of poor functional outcome and post-stroke cognitive impairment (PSCI) at 90 days [13]. Although several lines of evidence have supported an involvement of immunoproteasome in ischemia stroke and multiple sclerosis pathogenesis [14, 15], whether the immunoproteasome itself (such as LPM2) defects causes brain disease is still unclear. Therefore, the aim of this study was to explore the contribution of LMP2 in neurobehavioral functions in LPM2 gene knockout rats. The following issues would be addressed in the present study. First, the effect of LMP2 gene defect on neurobehavioral functions need to be clarified. Second, does LMP2 defect induce abnormalities in brain structure and cellular function? Third, what is the possible mechanism underlying these changes. Our study contributes to a further understanding of immuneproteasome deficiency leading to central nervous system abnormalities, which provides preliminary data for further research.

## Materials and methods

### Ethical approval and experimental animals

All experiments were approved by the Institutional Animal Ethical Committee of Fujian Medical University (No. FJMUIACUC2020-0059) (Animal license No. SYXK (Min) 2020–0005). LMP2 heterozygous type (LMP2^±^) Sprague–Dawley (SD) rats by use of CRISPR/Cas9 genome editing technology were purchased from Cyagen Biotechnology Co., LTD. (Animal License No. SCXK (Yue) 2018–0032). LMP2^±^ rats were bred in SPF grade animal room. They were housed five per cage to maintain social interaction. LMP2^±^ rats were mated. The tail genomic DNA of offspring rats were extracted and identified by polymerase chain reaction (PCR).The obtained homozygous rats were mated with the opposite sex heterozygous rats to obtain more homozygous rats with knockout LMP2 gene. Experiment rats aged 12-month-old were divided into two groups: LMP2 gene knockout (LMP2-KO) rats and littermate wild type (WT) rats (each group n = 15).

### Morris water maze (MWM)

The MWM system (RD1101-MWM, Mobiledatum Co., LTD., Shanghai, China) was used to evaluate spatial learning and reference memory as described previously [16, 17]. The lab room was kept dark and quiet during the test. During acquisition phase, each rat underwent four trials daily (10–15 min break between trials) for 5 consecutive days. Briefly, each trial started from a different location and lasted for 60 s. Each rat was trained to escape from the water by swimming to the hidden platform. Once the platform was found, the rat was allowed to stay on the platform for 10 s. If the rat could not locate the platform within 60 s, it would be guided to the platform and allowed to stay there for 15 s (assigned a latency of 60 s). On the sixth day, the probe test of platform location memory retention was conducted with the platform removed. Rats were placed into the water from the opposite side of the original platform quadrant and were allowed to swim freely for 60 s to find the location of the original platform. All parameters including the latency and path length to platform, the time spent in the target quadrant, average swimming speed, and the numbers of crossings over the platform site were calculated using system software (Mobile datum Co., LTD., Shanghai, China).

### Open field test (OFT)

The OFT was performed to assess locomotor activity and anxiety-like behavior according to a previous report [18]. Briefly, the test was conducted for 5 min in a dimly lit room. The box system consists of an arena measuring 100 cm × 100 cm × 40 cm (L × W × H). The arena was made of black high-density polyethylene panels that were fastened together. A video tracking system was suspended over the box to record the rats’ movements’ orbits. The rats were placed in the center of arena and allowed to freely explore the area for 5 min. The total traveled distance, distance and the time in the central area of each rat were calculated.

### Elevated plus maze (EPM)

The elevated plus maze test was applied to assess anxiety-like behavior as described previously [19]. A decrease in the open arm activity (duration and/or entries) reflects anxiety-related behaviors. The system consists of four black polypropylene arms, including two opposing open arms (50 cm × 10 cm × 0.5 cm), two opposing closed arms (50 cm × 10 cm × 40 cm) and a central platform (10 cm × 10 cm) and elevated 50 cm above the ground. The test was performed in a quiet, dimly lit room. Rats were placed at the central platform facing an open arm and allowed 5 min to explore the maze. Their behavior was recorded with a camera mounted above the maze. The entries into the open arms and duration in the open arms were calculated.

### Luxol fast blue (LFB) staining

LFB is widely used to detect demyelination in the CNS. Coronal Sects. (20 μm) were stained with Luxol Fast Blue Stain Kit according to the manufacturer’s instructions (Abcam, USA) and conducted as described previously [20]. The severity of the white matter lesions was graded as described [21].

### Evaluation of BBB Permeability

Evans blue (EB) dye (Sigma-Aldrich, USA) extravasation and FITC-dextran cerebral fluorescent angiography were used to evaluate BBB permeability using spectrophotometry and fluorescence microscopy as described previously [22–24].

### Blood routine and blood biochemicalexamination

After the animals were deeply anesthetized, the thoracic cavity was exposed, and 5 ml of blood was drawn from the right atrial vein with a sterile syringe. Blood routine and blood biochemical examination were conducted by the biotech company of Zolgene Biotech, Inc. (Fuzhou, China).

### Immunofluorescence (IF)

Immunofluorescence was performed as described previously [22]. After blocking with 10% normal goat serum (Sigma-Aldrich, USA), slices were incubated with primary antibodies as following: rabbit anti-GFAP (1:300; Cell Signaling Technology, USA), rabbit anti-IBA1 (1:400; Abcam, Cambridge, MA, USA), rabbit anti-beta Amyloid (1:50; Abcam, Cambridge, MA, USA), and mouse anti-Olig1 (1:100; Santa Cruz, USA). After incubated overnight at 4 °C and washed three times in 0.01 M PBS (3 × 5 min), slices were incubated with the following secondary antibodies for 1 h at room temperature: Alexa Fluor^®^ 594 conjugated goat anti-rabbit IgG or Alexa Fluor^®^ 488 conjugated goat anti-rabbit IgG or Alexa Fluor^®^ 488 conjugated goat anti-mouse IgG(1:1000; Cell Signaling Technology). Slices were mounted in ProLong^®^ Gold antifade reagent (Thermo Fisher Scientific, USA) prior to imaging.

### Western blotting

Approximately 30 μg of total protein was loaded on an SDS–polyacrylamide gel as described previously [22]. The primary antibodies were used as follows: rabbit anti-myelin basic protein (MBP) and rabbit anti-IRAK1 (1:1000; Abcam, Cambridge, MA, USA), mouse anti-IL-6 (1:500; Abcam, Cambridge, MA, USA), mouse anti-Olig1 and mouse anti-TNF-a (1:300; Santa Cruz, USA), mouse monoclonal anti-β-actin (1:3000; Cell Signaling Technology, USA). Membranes were incubated with the secondary antibodies for 1 h at room temperature: horseradish peroxidase (HRP)-conjugated goat anti-rabbit or HRP-conjugated goat anti-mouse IgG antibody (1:3000; Cell Signaling Technology, USA). The bands were quantified by densitometry with ImageJ software (ImageJ 1.4, NIH, USA).

### Statistical analysis

All datawere analyzed using SPSS 19.0 software (version 19, IBM Corp., Armonk, NY, USA) and are presented as the mean ± standard deviation. The water maze escape latency and the path length were analyzed using repeated measures two-way ANOVA. Other parametric data from different groups were compared using one-way ANOVA. The least-significant difference post hoc test was used for comparison within groups. GraphPad Prism 8.0 (GraphPad Software Inc., La Jolla, CA, USA) was used to make statistical graph. A value of P < 0.05 was considered statistically significant.

## Results

### Comparison of basal parameters between the WT and LMP2-KO rats

**Fig. 1:**
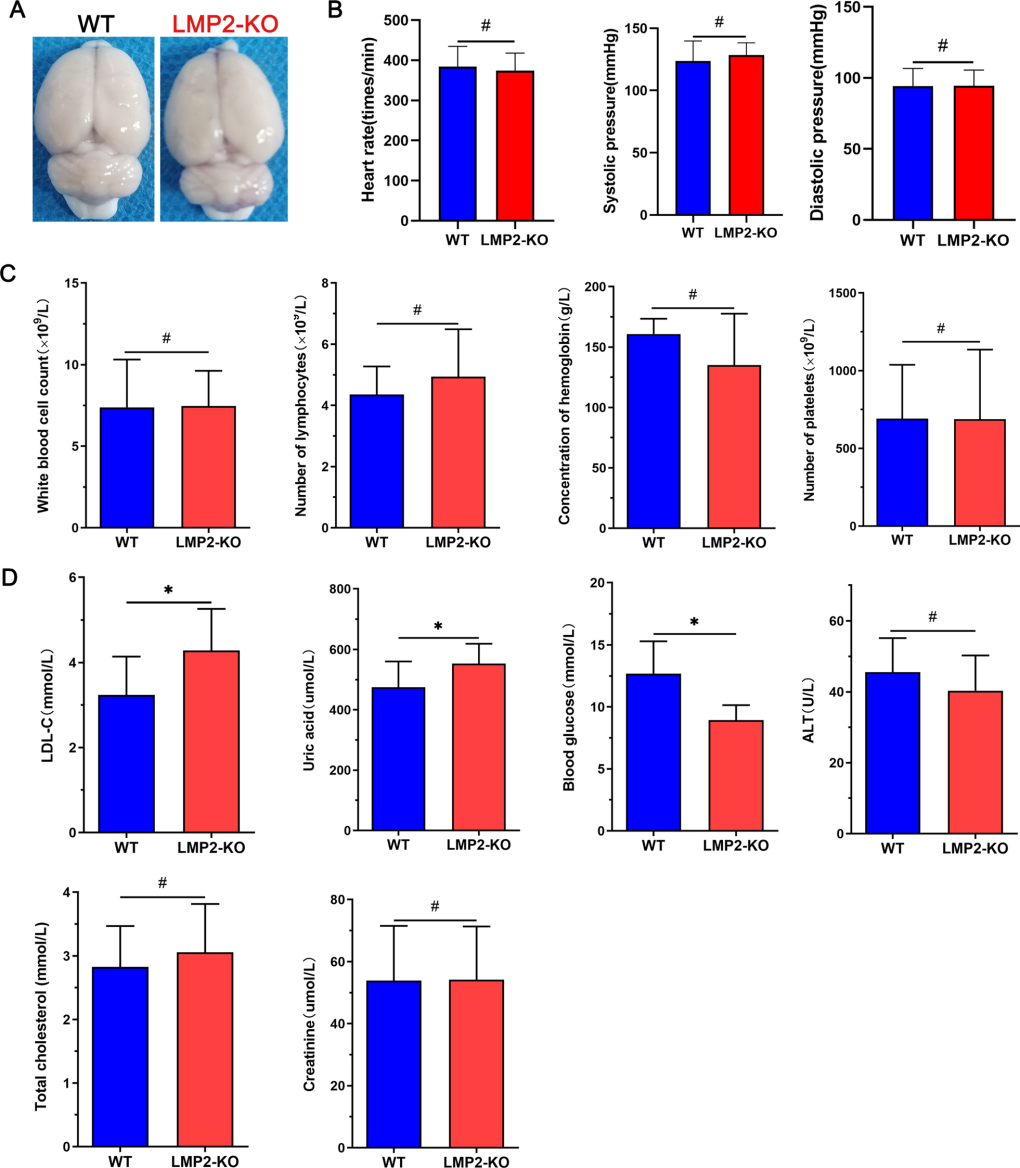
Comparison of basal parameters between the LMP2-KO and WT rats. **A** There was no significant difference in gross brain appearance between the LMP2-KO and WT rats. **B** There were no significant differences in heart rate and blood pressure between the two groups (^#^P > 0.05). **C** The levels of ALT, total cholesterol, LDL-C, blood glucose, creatine and uric acid from blood samples of LMP2-KO and WT rats were comparison, and there were significant increased levels of blood LDL-C, uric and glucose in the LMP2-KO rats compared with the WT rats (^#^P > 0.10,*P < 0.05). (**D**) Blood biochemical examination showed that the levels of low-density lipoprotein cholesterol (LDL-C), uric acid (Uric) and blood glucose in the LMP2-KO group were higher than those in the WT group, respectively (*P < 0.05)

First, there was no significant difference of daily variability in feeding, drinking, and movement behaviors, growth and development between the two groups of rats. Besides, it seemed no obviously difference in gross brain appearance between the WT and LMP2-KO rats (Fig. 1A). Furthermore, LMP2 gene deletion did not result in significant changes in heart rate and blood pressure compared with the WT group (Fig. 1B). In addition, blood routine examination indicated there were no comparable in white blood cell count, lymphocyte count, hemoglobin concentration and platelet count between the two groups (Fig. 1C). Interestingly, blood biochemical examination showed that the levels of low-density lipoprotein cholesterol (LDL-C), uric acid (Uric) and blood glucose in the LMP2-KO group (LDL-C:4.29 ± 0.98 mmol/L; Uric:552.96 ± 65.79 umol/L; GLU 12.68 ± 2.62 mmol/L) were higher than those in the WT group (LDL-C: 3.24 ± 0.89 mmol/L; Uric: 474.80 ± 85.61 umol/L; GLU 8.94 ± 1.21 mmol/L), respectively (*P < 0.05); but there were no significant differences in alanine aminotransferase (ALT), total cholesterol and creatinine levels between the two groups (Fig. 1D) (Table1) (^#^P > 0.10,*P < 0.05).

**Table 1:** Comparison of basal parameters between the WT and LMP2-KO rats

Item	WT rats	LMP2-KO rats	P value	Item	WT rats	LMP2-KO rats	P value
Heart rate	374.47 ± 43.65	384.42 ± 50.49	0.574	LDL-C (mmol/L)	3.24 ± 0.89	4.29 ± 0.98	0.023
Systolic pressure (mmHg)	123.67 ± 16.14	128.43 ± 9.85	0.350	Uric acid (umol/L)	474.80 ± 85.61	552.96 ± 65.79	0.034
Diastolic pressure (mmHg)	94.33 ± 12.35	94.42 ± 11.17	0.983	Blood glucose (mmol/L)	8.94 ± 1.21	12.68 ± 2.62	0.001
White blood cell count (× 10^9^/L)	7.38 ± 2.94	7.46 ± 1.16	0.962	ALT (U/L)	45.57 ± 9.60	40.34 ± 9.92	0.246
Number of lymphocytes (× 10^9^/L)	4.36 ± 0.91	4.94 ± 1.55	0.492	Total cholesterol (mmol/L)	2.82 ± 0.64	3.05 ± 0.76	0.478
Concentration of hemoglobin (g/L)	160.80 ± 12.68	135.20 ± 42.45	0.232	Creatinine (umol/L)	53.89 ± 23.70	54.14 ± 22.30	0.975
Number of platelets (× 10^9^/L)	690.60 ± 347.83	688.60 ± 446.56	0.994				

### LMP2 gene knockout results in cognitive impairment in rats

**Fig. 2:**
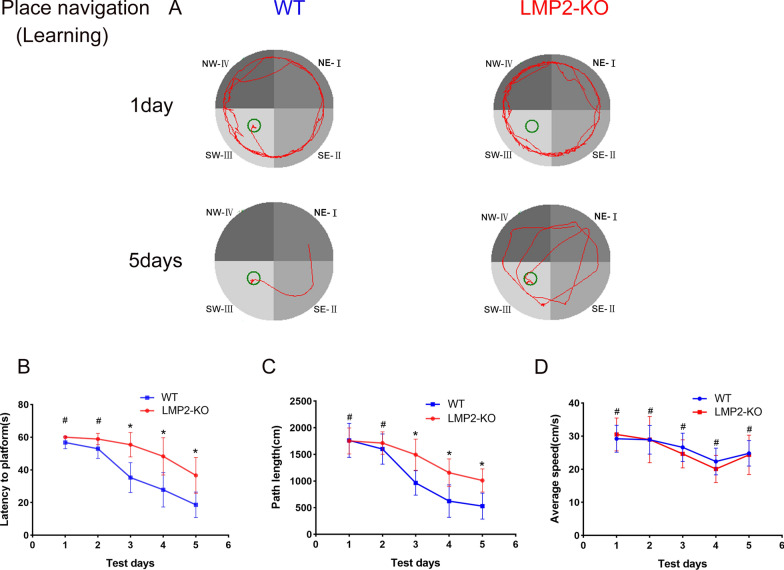
LMP2 defect increases the latency and path length to find the hidden platform in place navigation. LMP2-KO and WT rats were subjected to Morris water maze test (MWM) for place navigation for 5 consecutive days. The escape latency and the path length were analyzed using repeated measures two-way ANOVA. **A** Representative track plots of MWM for place navigation. **B** The latency significantly increased in the LMP2-KO rats as compared with the WT rats. **C** The path length of finding the hidden platform significantly increased in the LMP2-KO rats as compared to the WT rats. **D** The average swimming speed was not significantly different between the LMP2-KO and the WT rats (^#^P > 0.10,*P < 0.05)

As shown in Fig. 2, there was no significant difference in the latency to platform and path length during the initial 2 days of acquisition/learning period between the WT group and the LMP2-KO group. However, with the extension of training time, rats in the WT group found the platform position earlier than those in the LMP2-KO group, and LMP2-KO rats spent more time and longer path length looking for a platform, compared to the WT controls from the 3rd day to the 5th day in the acquisition/learning phase. For example, on the 5th day of training, the latency to platform and path length before finding the platform in the LMP2-KO group was significantly higher than those in the WT group, respectively. However, there was no significant difference in the average swimming speed between the two groups during the 5 days training period (Fig. 2) (^#^P > 0.10,*P < 0.05).

**Fig. 3:**
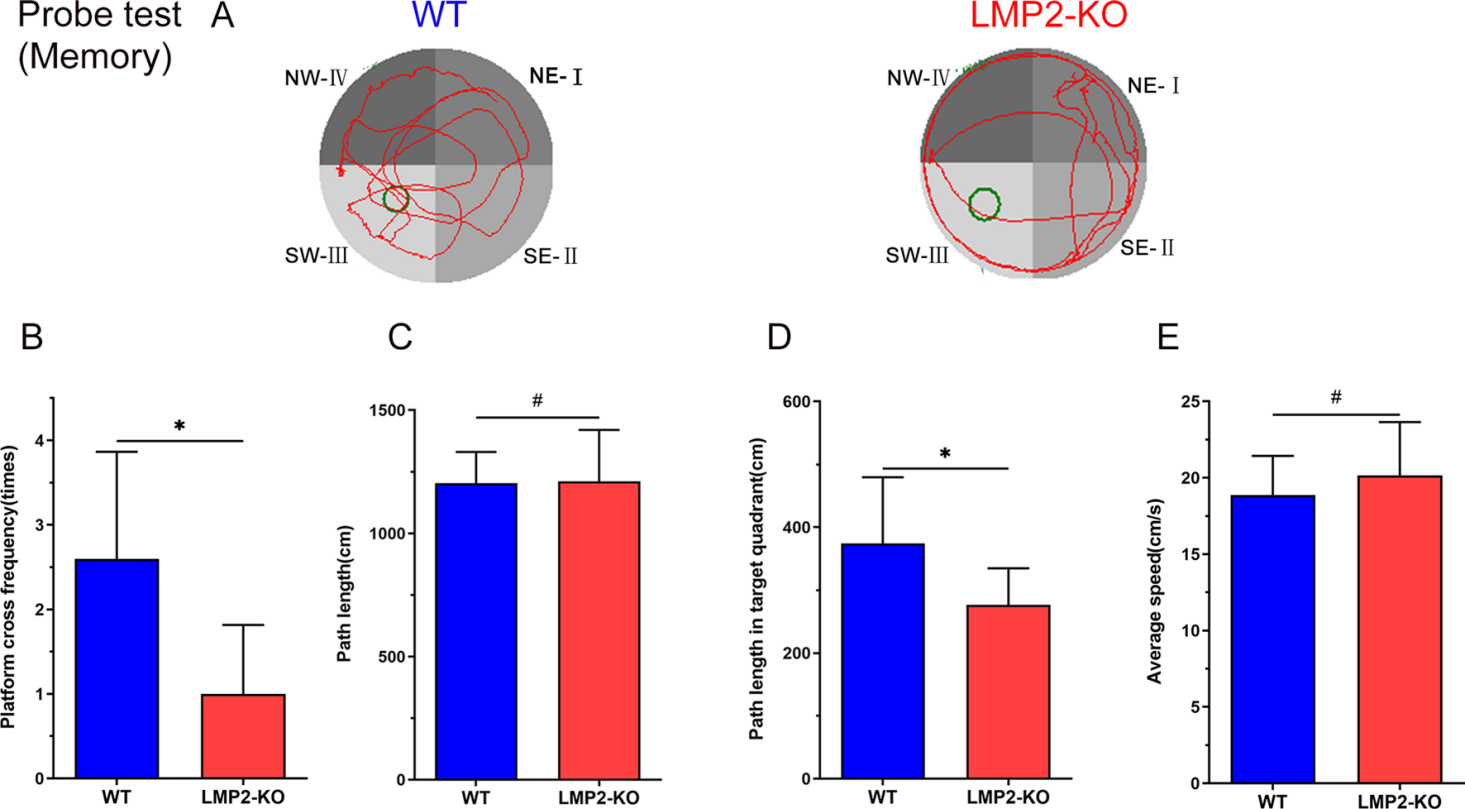
LMP2 defect decreases the frequency over the target quadrant in probe test. Both LMP2-KO and WT rats were subjected to Morris water maze test (MWM) for probe tests on the 6th day followed place navigation. The data were analyzed using one-way ANOVA. **A** Representative track plots of MWM for probe tests. **B** The frequency crossing through the platform significantly lower in LMP2-KO rats than in WT rats (*P < 0.001). **C** The total path length and **D** the percentage of path length in target quardrant and **E** the average swimming speed was compared between LMP2-KO and WT rats, respectively (^#^P > 0.10,*P < 0.05)

In the probe test, as shown in Fig. 3, the frequency of crossing over the platform was significantly lower in the LMP2-KO group than that of the WT group (P < 0.05). Although the data demonstrated that there was no significant difference in the total path length between the two groups, the path length in the target quadrant in the LMP2-KO group was shorter than that in the WT group. In addition, there was no significant difference in the average swimming speed between the two groups (Fig. 3) (^#^P > 0.10,*P < 0.05).

### Deletion of the LMP2 gene reduces rats’ exploratory activities.

**Fig. 4:**
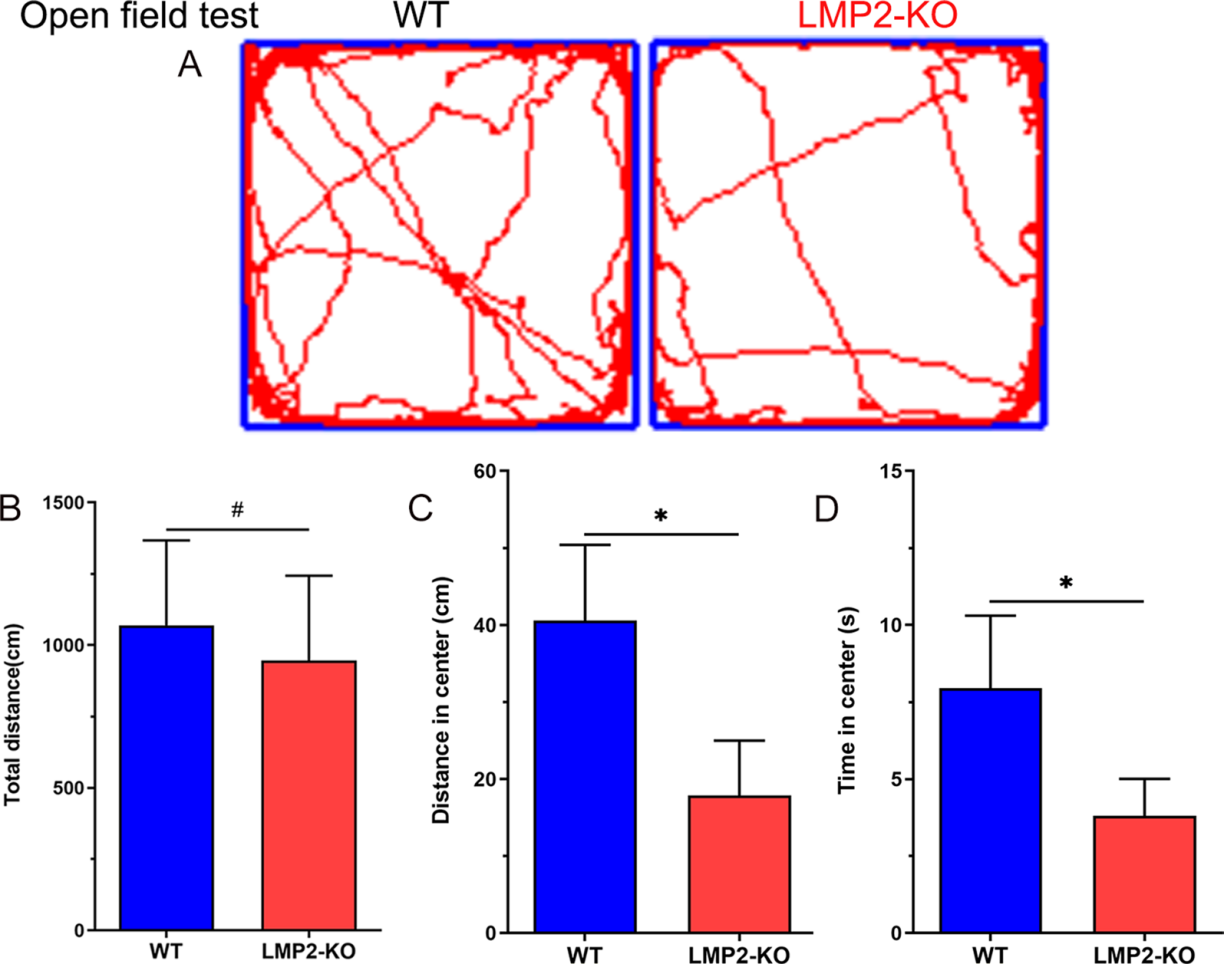
Deletion of the LMP2 gene reduces rats’ exploratory activities. **A** Representative tracks for open field test. **B** There was no significant difference in the total distance between the WT rats and the LMP2-KO rats (^#^P > 0.10). **C–D** Compared with the WT rats, both the distance traveled in center area and the time spent in the center were significantly lower in the LMP2-KO rats, respectively (*P < 0.001)

As shown in Fig. 4, the results showed that, there was no comparable in the total distance traveled during five minutes between the WT and LMP2-KO rats (^#^P > 0.05). However, compared with the WT rats, both the distance traveled in center area and the time spent in the center were significantly lower in LMP2-KO rats, respectively (Fig. 4) (*P < 0.001).

### Deletion of the LMP2 gene increases rats’ anxiety-like behavior

**Fig. 5:**
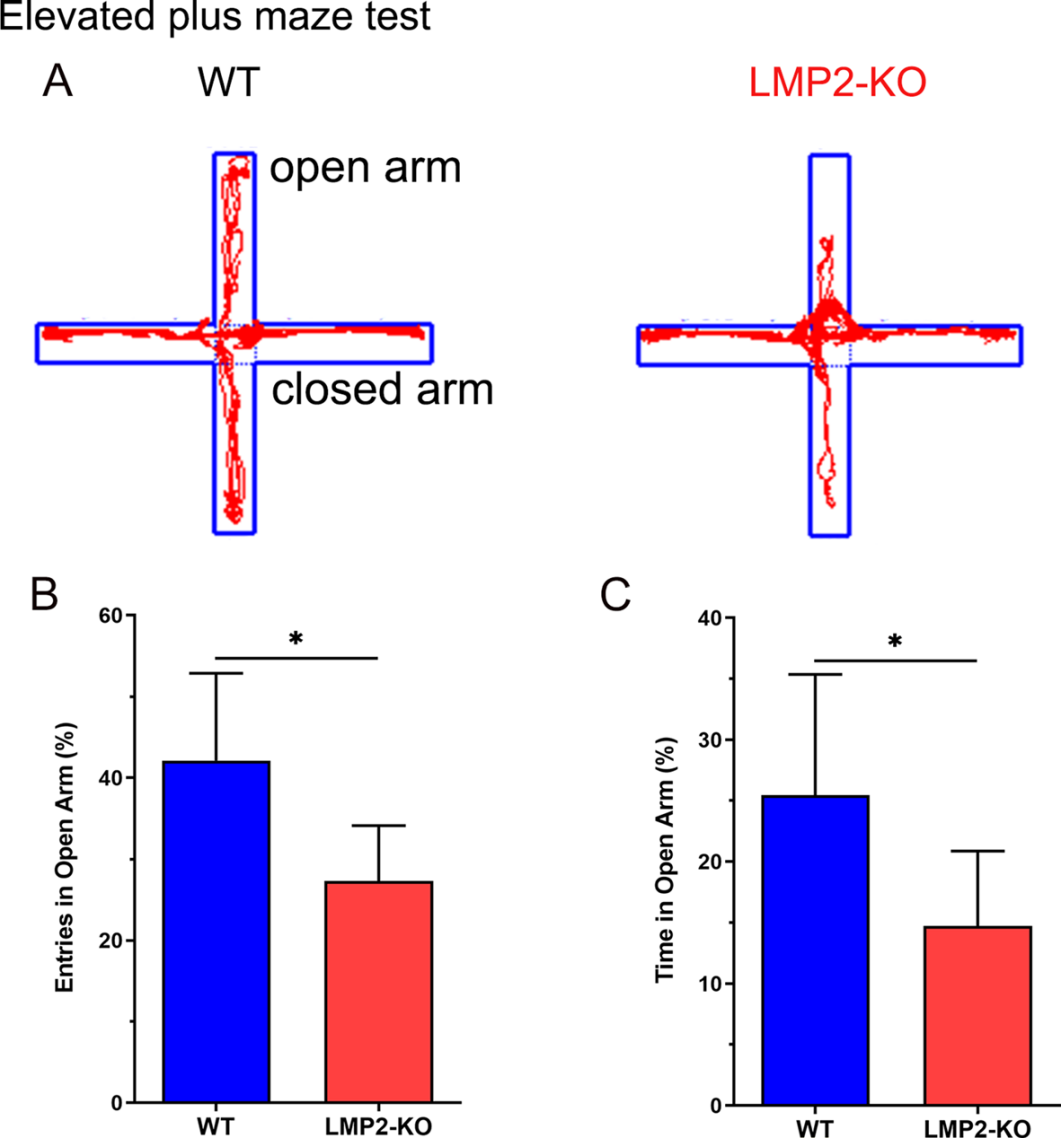
Deletion of the LMP2 gene increased rats’ anxiety-like behavior measured by elevated plus maze. **A** Representative tracks for the elevated plus maze test. **B**–**C** Compared with the WT rats, both the percentages of the entries in open arms and the time spent in open arms were significantly less in the LMP2-KO rats than that in WT rats, respectively (*P < 0.05)

The above preliminary data suggested that LMP2-KO rats seemed to have anxiety-like behavior. Next, we used elevated plus maze experiment to further assess whether these rats showed signs of anxiety. As shown in Fig. 5, WT rats repeatedly walking between the open and closed arms, but LMP2-KO rats preferred to walk in the closed arms, less chance to entry to the open arms. Compared with the WT rats, both the percentages of the entries in open arms and the time spent in open arms were significantly less in LMP2-KO rats than those in WT rats, respectively (Fig. 5) (*P < 0.05).

### LMP2 gene deficiency results in myelin loss and remyelination coexisted in brain of rats

**Fig. 6:**
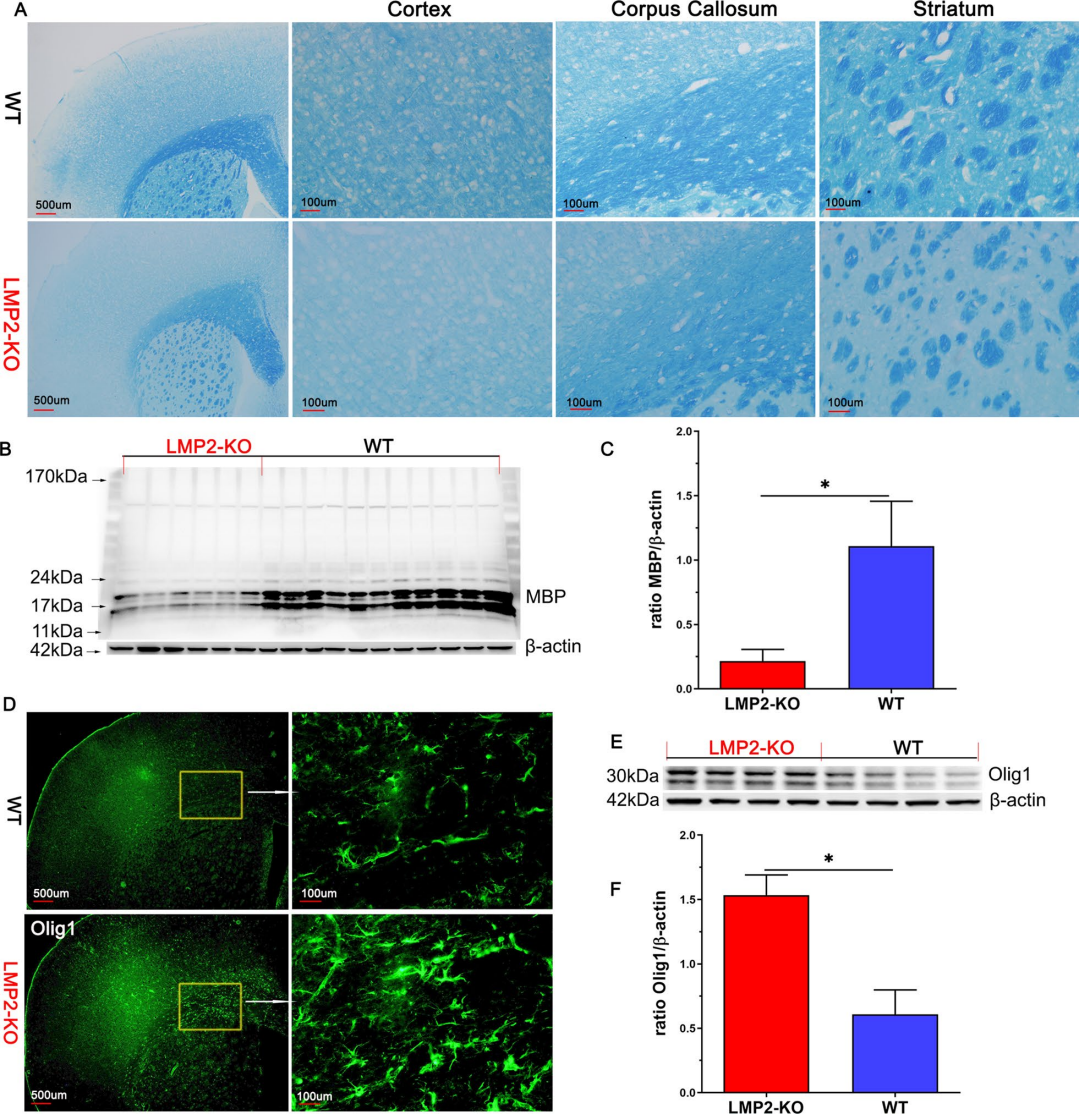
LMP2 gene deficiency results in myelin loss and remyelination coexisted in brain of rats. **A** LFB staining showed myelin loss of the cortex, corpus callosum and striatum of WT, LMP2-KO rats, respectively. **B**–**C** Representation image of MBP protein expression detected by western blotting and quantification data. **D**–**F** Immunofluorescence and western blotting showed that increased protein expression of oligodendrocytes-1(Olig1) in the LMP2-KO rats compared with the WT rats. Scale bars: 500 μm and 100 μm. *P < 0.001

Our previous study suggested that white matter demyelization accompany with cognitive impairment in eNOS deficiency mice [25]. Therefore, we were interested to explore whether there exists similar phenomenon in brain of LMP2 gene deficiency rats. As shown in Fig. 6A, LFB staining showed severe myelin loss of cerebral cortex, corpus callosum and striatum in the LMP2-KO rats compared with the WT rats, respectively. These finding were also supported by measuring the protein levels of MBP detected by western blotting. MBP protein expression in the LMP2-KO group was further decreased compared with that in the WT group (P < 0.001) (Fig. 6B, C).

OLIG1, a member of the oligodendrocyte lineage-specific basic helix-loop-helix (OLIG) family of transcription factors, is a gene regulator and expressed in oligodendrogenesis which are the source cells of myelination in the central nervous system (CNS). To our surprise, we observed demyelination and remyelination coexisted in brain of LMP2-KO rats. Both IF and western blotting showed that increased protein expression of oligodendrocytes-1(Olig1) in forebrain of LMP2-KO rats compared with the WT rats (P < 0.001) (Fig. 6D–F).

### LMP2 gene deficiency significantly induces BBB leakage and decreases tight junction proteins expression compared to the WT rats

**Fig. 7:**
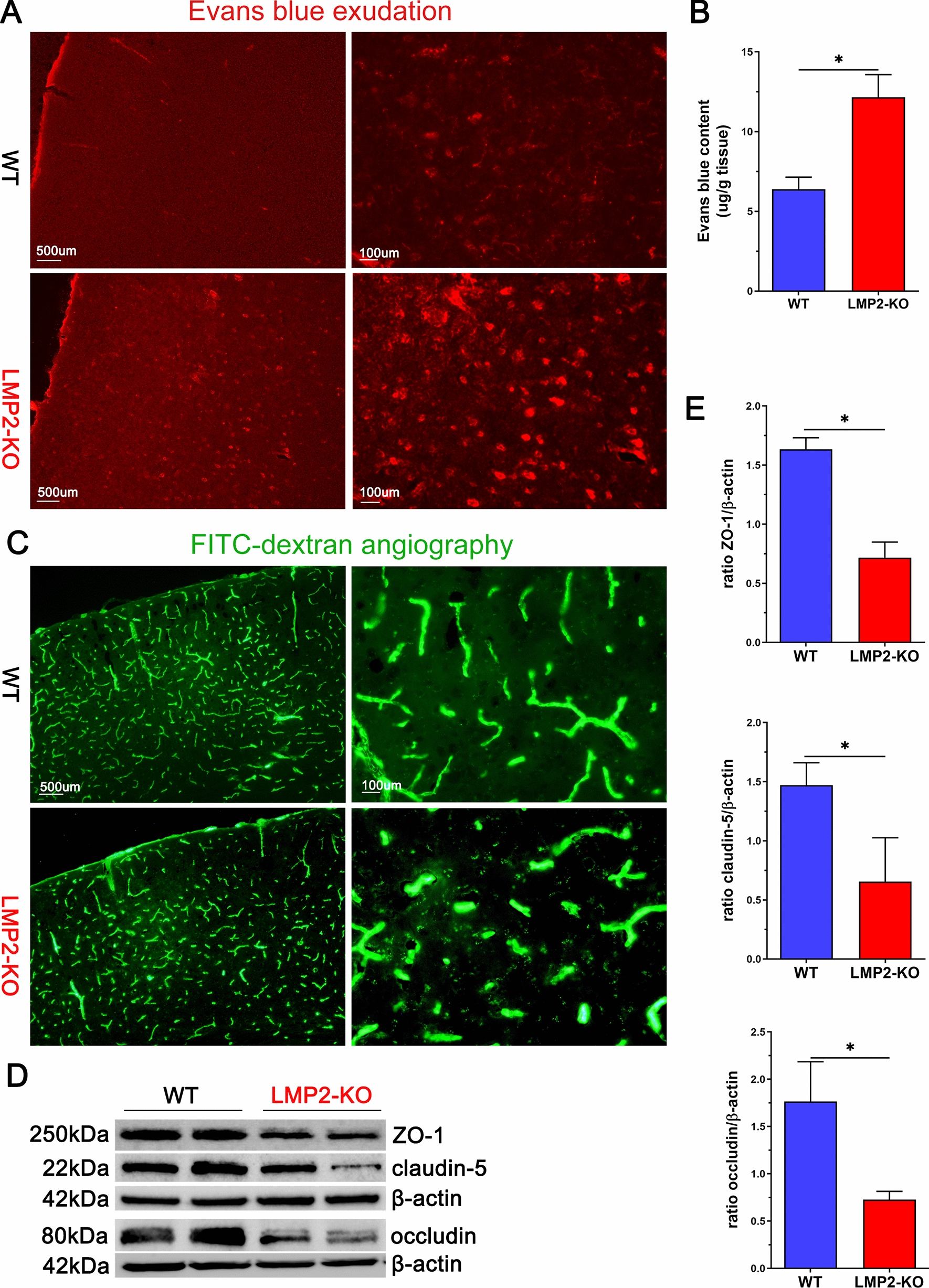
LMP2 gene deficiency significantly induces BBB leakage and decreases tight junction proteins expression compared to the WT rats. **A**–**B** Evans blue (EB) exudation was observed under fluorescence microscope and quantificated, respectively. The level of EB exudation significantly increased in the LMP2-KO group than that in the WT group. **C** FITC-dextran angiographic micrographs indicated the tracer was confined to the capillaries in wild-type littermates, whereas LMP2-KO rats showed large amounts of tracer leakage in the brain parenchyma. **D**–**E** Representation images of tight junction proteinsZO-1, claudin-5 and occluding expression detected by western blotting and quantification data. Scale bars: 500 um; 100 μm.*P < 0.001

Recent evidence has suggested that BBB disruption is an early biomarker of human cognitive impairment, including the early clinical stages of AD and vascular cognitive impairment [26]. Our previous work confirmed the correlation between LMP2 and BBB dysfunction under ischemia stroke conditions [14, 27]. To our surprise, LMP2 gene deletion led to BBB leakage indicated by Evans blue (EB) exudation evaluation. A small amount of EB dye was observed to exude outside the blood vessels in the brain of WT rats under fluorescence microscope and further supported by quantification of EB content in the cerebral of rats. However, EB exudation was significantly increased in the LMP2-KO group than that in the WT group (*P < 0.001) (Fig. 7A, B). Similarly, FITC-dextran angiographic micrographs indicated the tracer was confined to the capillaries in wild-type littermates, whereas LMP2-KO rats showed large amounts of tracer leakage in the brain parenchyma (Fig. 7C). The BBB which consists of brain endothelial cells interconnected by tight junctions is essential for the homeostasis of the CNS. Tight junctions are consisted of a number of proteins, including zonula occludens-1 (ZO-1), occluding and claudin-5. LMP2 deficiency significantly decreases the protein levels of ZO-1, claudin-5 and occluding detected by western blotting (*P < 0.001) (Fig. 7D, E).

### LMP2 gene deficiency enhances Amyloid-β protein deposition compared to the WT rats

**Fig. 8:**
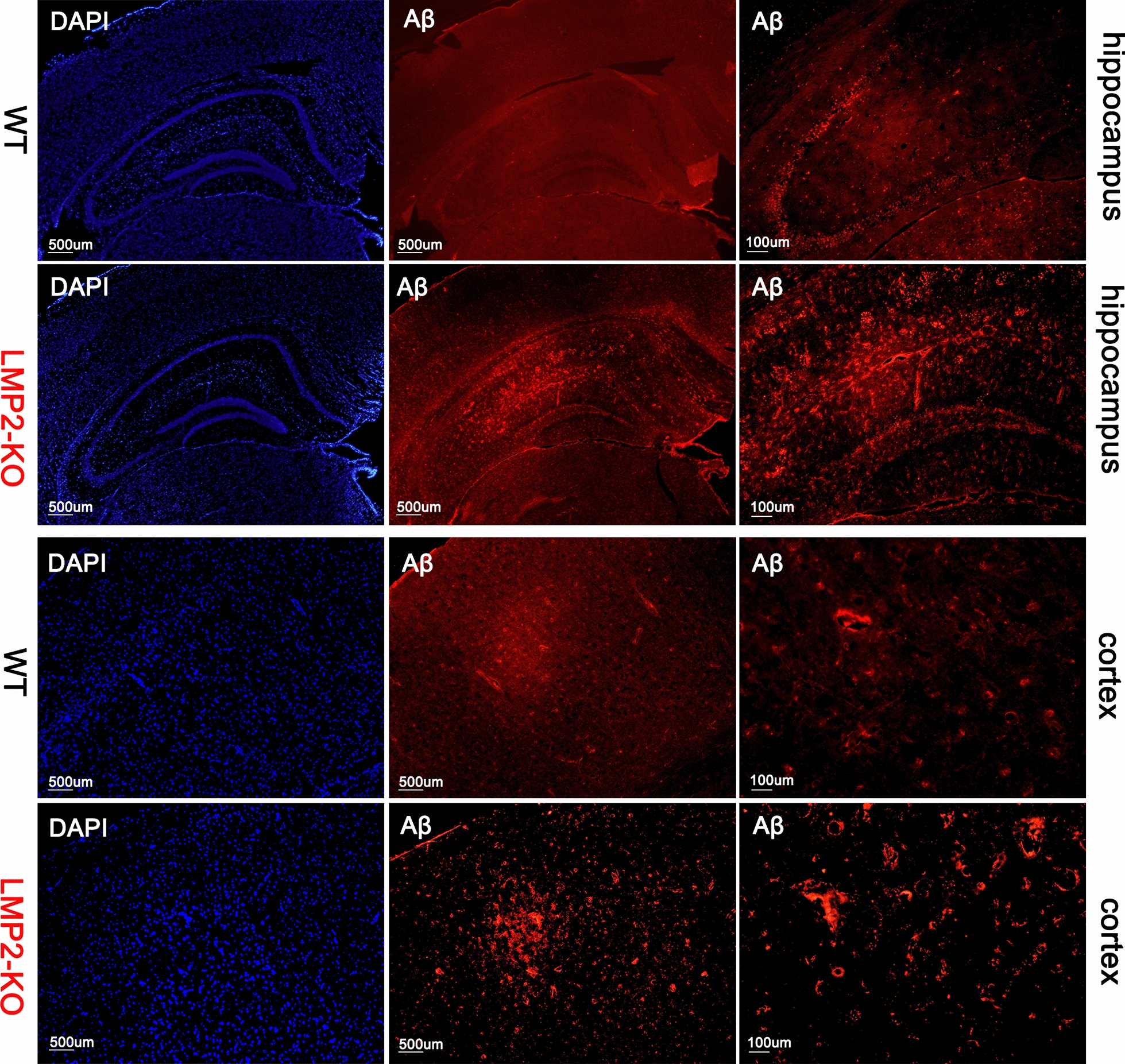
LMP2 gene deficiency enhances Amyloid-β protein deposition compared to the WT rats. LMP2 deficiency significantly enhanced Aβ protein deposition. Immunofluorescence showed that there was more Aβ protein deposition in the hippocampus and cerebral cortex of LMP2 gene deficiency rats compared with the WT rats. Scale bars: 500 um; 100 μm

The BBB dysfunction induces the failure of amyloid-β (Aβ) protein transport from brain to the peripheral circulation across the BBB and involves in the pathogenesis of Alzheimer's disease (AD) [28]. In addition, increasing evidence has suggested that immunoproteasome participates in the pathology of AD [29]. Interestingly, we observed that LMP2 deficiency significantly enhanced Aβ protein deposition. Immunofluorescence showed that there was more Aβ protein deposition in the hippocampus and cerebral cortex of LMP2 gene deficiency rats compared with the WT rats (Fig. 8).

### LMP2 deficiency significantly enhances oxidative stress, reactive glia and proinflammation compared to the WT rats

**Fig. 9:**
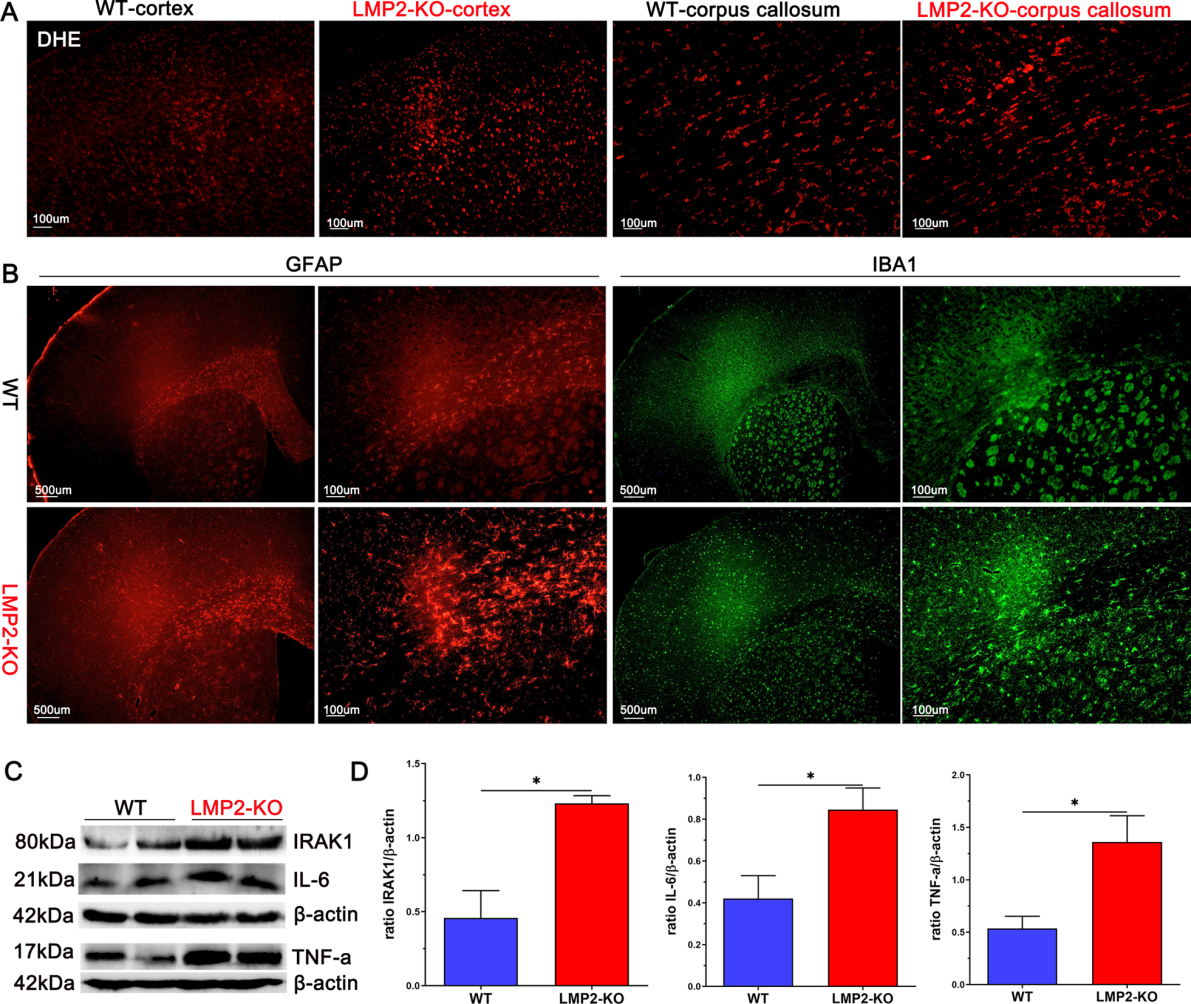
LMP2-KO rats exhibit significantly enhanced oxidative stress, increased expression of astrocyte, microglial expression and inflammation compared to WT rats, respectively. **A** DHE staining showed ROS levels in the cortex and corpus callosum of WT and LMP2-KO rats, respectively. **B** Expression of astrocytes (GFAP), microglias (IBA1) in brain of WT and LMP2-KO rats, respectively. **C**–**D** Representation images of IRAK1, IL-6 and TNF-a proteins expression detected by western blotting and quantification data. Scale bars: 500 um; 100 μm. *P < 0.001

The immunoproteasome participates in the regulation of oxidative stress and neuroinflammation under different context [3, 30]. Importantly, oxidative stress and inflammation are strongly related to a variety of neurological diseases, including stroke, AD and Parkinson's disease (PD). Interestingly,LMP2-KO rats exhibited significantly enhanced oxidative stress, as shown in Fig. 9A, compared to the WT rats, the levels of ROS, as indicated by DHE fluorescence staining, were markedly elevated in the cerebral cortex and corpus callosum of LMP2-KO rats.

In addition, immunofluorescence showed that increased expression of astrocytes and microglials, labeled by GFAP and IBA1, respectively (Fig. 9B). Gliosis leads to the activation of inflammation-related signaling pathway and releases proinflammatory cytokines. As shown in Fig. 9C, D, the levels of IRAK1, IL-6 and TNF-a proteins expression detected by western blotting were markedly upregulated in the LMP2-KO group compared with the WT group, respectively (*P < 0.001) (Fig. 9C, D).

## Discussion

The major finding of this study is that LMP2 gene global deletion caused significant neurobehavioral dysfunctions including cognitive impairment and decreased exploratory activities, increased anxiety-like behavior. Along with this, the signs of metabolic abnormalities including higher levels of LDL, uric acid and blood glucose, multiple myelin loss, elevated levels of ROS, increased BBB leakage and enhanced amyloid-β protein deposition were obviously observed in the LMP2-KO rats. All these factors maybe interact with each other and eventually led to chronic oxidative stress and neuroinflammation response in the brain regions of LMP2-KO rats, which contributed to the initial and progress of cognitive impairment.

Although the biological importance roles of the ubiquitin proteasome system (UPS) in the control of myriad cellular processes have been well documented, the significance of the immunoproteasome has not been well comprehended until now. The immunoproteasome possesses broader biological roles including immune and non-immune functions attributed to its three immunosubunits β1i, β2i and β5i [2]. Particularly, LMP2 has recently drawn considerable attention in many studies. Structurally, LMP2 is a critical component for proteasome activity in that LMP2 is essential for the proper incorporation of the immunoproteasome [31]. LMP2 is expressed in neuron, astrocyte, microglia and endothelial cells in brain tissue of human or rodent animal [3, 9, 32]. For example, LMP2 were increased expression in brain areas affected by AD or Huntington's disease which often displays cognitive dysfunction [9, 33]. However, it is not clear whether LMP2 change is the cause or result of these neurodegenerative diseases. Interestingly, in this study, we provided new evidence that LMP2 gene deletion resulted in cognitive dysfunction, reduced rats’ exploratory activities, increased rats’ anxiety-like behavior and without strong effects on gross locomotor abilities (such as swimming speed). Similarly, a recent reported that LMP7-deficient mice expressed more anxiety and increased cued fear and no strong effects on gross locomotor abilities [12]. Taken together, these data suggest that the immunoproteasomeis closely related to cognitionand emotional behavior. However, the related mechanism underlie this remains unclear. Recent reported that immunoproteasome inhibitor ONX-0914 affected long-term potentiation in murine hippocampus, a form of synaptic plasticity thought to contribute to learning and memory [34].Induction and maintenance of long-term potentiation is directly dependent on selective targeting of proteins for proteasomal degradation. Therefore, we postulated one of possible mechanisms is that the immunoproteasome plays an important role in synaptic plasticity which underlie learning and memory processes [35].

Several factors are related with the development and progress of cognitive impairment, including abnormal metabolism of lipids and glucose [26, 36], BBB damage [37], mitochondrial dysfunction [38, 39], white matter demyelination [25, 40]. For example, BBB injury induces the failure of Aβ proteins transport from brain to the peripheral circulation across the BBB, and eventually Aβ proteins aggregate in the brain further aggravate BBB damage, forming a vicious cycle [28, 41]. This phenomenon can also be seen in the present study, we observed that Aβ proteins deposition in the hippocampus and cerebral cortex of LMP2-KO rats compared with the WT rats. Interestingly, it seems that oxidative stress and inflammation reaction are the two leading causes for these changes. Brain neurons are sensitive to oxidative stress and inflammation damage. Indeed, oxidative stress and inflammation response are closely together with each other and are involved in the pathogenesis of stroke and neurodegenerative diseases [42, 43]. Alongside immune functions, immunoproteasome play a crucial role in removing oxidant-damaged proteins and protect cell viability against oxidative stress [1, 44]. In the absence of LMP2, accumulation of oxidized and polyubiquitinated proteins were observed in the liver and brain of LMP2-KO mice [8]. In line with this observation, LMP2-KO rats exhibited significantly enhanced oxidative stress; the levels of ROS indicated by DHE fluorescence staining were markedly elevated in the cerebral cortex and corpus callosum of LMP2-KO rats. In addition, our previous study found that inhibition of LMP2 decreased the protein levels of IL-1β and TNF-α in rat stroke model [3]. However, to our surprise, LMP2 gene deletion led to the augmentation of neuroinflammation accompanied with upregulation protein expressions of IL-1R-associated kinase 1 (IRAK1), IL-6 and TNF-a. Notably, IRAK1 is a key signaling mediator in the TLR/IL-1R signaling pathway which initiates a cascade of diverse downstream proinflammatory events [45]. Astrocyte and microglia can be polarized to proinflammatory phenotype by increasing IRAK1 expression and induce inflammation response [46]. Taken together, this evidence supports the view of LMP2 deficit promoting oxidative stress and inflammation response, but a great deal of work need to do insight into the detail molecular mechanisms underlying this.

In the present study, LFB staining showed severe myelin loss of cerebral cortex, corpus callosum and striatum in the LMP2-KO rats compared with the WT rats. Besides, the change of myelin loss was also confirmed by MBP protein expression decreased in the brain tissue of LMP2-KO rats. Unexpectedly, we observed demyelination and remyelination coexisted in the LMP2-KO rats. In fact, dynamics of myelin loss and generation can be observed in many CNS diseases, particularly in multiple sclerosis (MS). The innate immune response contributes to promoting remyelination observed in clinical MS disease [47]. However, despite this increase, overall levels of myelination indicated by MBP protein expression were decreased in the brain tissue of LMP2-KO rats. We assumed this kind of endogenous remyelination would not be enough to compensate the lost myeline and reverse the symptoms associated with demyelination and axonal death. Recent reported that genetically or pharmacologically enhancing myelin renewal could improve the memory-related tasks performance of APP/PS1 AD model mice [40]. In summary, this evidence suggests the potential of enhancing myelination maybe as a promising therapeutic strategy to improve cognitive impairment.

The present study has some limitations. First, we observed neurobehavioral abnormalities in the LMP2 knockout rats at a specific age (12 months old), but did not further explore the age at which these rats began to develop neurobehavioral abnormalities. Second, we did not administrate some therapeutic approach involves increasing the endogenous antioxidant activity and/or reducing ROS production as well as anti-inflammatory drug to treat these experimental animals, which would provide supplemental data to support the contribution of oxidative stress and inflammation response involved in the mechanisms of neurobehavioral abnormalities in rats. In addition, this study explored the phenomenon of LMP2-KO in rats, but we did not investigate its potential profound mechanisms. Cognitive impairment has been linked to several factors, including the excessive formation of ROS, mitochondrial dysfunction, inflammation and oxidative damage [38, 48]. Especially, whether mitochondrial abnormalities occurred in these knockout rats requires further investigation in the future.

## Conclusion

Our study demonstrates for the first time that LMP2 gene global deletion resulted in cognitive impairment and decreased exploratory activities, increased anxiety-like behavior. Chronic oxidative stress and inflammation response in the brain regions of LMP2-KO rats maybe work together and lead to the initial and progress of neurobehavioral dysfunctions. Future studies to reveal the detail molecular mechanisms underlying this are warranted.

## Data Availability

The datasets used and/or analyzed during the current study are available from the corresponding author on reasonable request.

## Abbreviations

BBB: Blood–brain barrier
LMP2: Low molecular mass peptide 2
MECL-1: Multicatalytic endopeptidase complex-like 1
ZO-1: Zonula occluden-1
CNS: Central nervous system
UPS: Ubiquitin proteasome system
ROS: Reactive oxygen species
Aβ: Amyloid-β
AD: Alzheimer's disease
MS: Multiple sclerosis
TNF-α: Tumor necrosis factor-α
IL-1β: Interleukin-1β
IRAK1: IL-1R-associated kinase 1
MMPs: Matrix metalloproteinases
MMP-2: Matrix metalloproteinases-2
LFB: Luxol fast blue
DHE: Dihydroethidium

## References

[1] Seifert U, Bialy LP, Ebstein F, Bech-Otschir D, Voigt A, Schroter F, Prozorovski T, Lange N, Steffen J, Rieger M (2010). Immunoproteasomes preserve protein homeostasis upon interferon-induced oxidative stress. Cell.

[2] Angeles A, Fung G, Luo H (2012). Immune and non-immune functions of the immunoproteasome. Front Biosci.

[3] Chen X, Zhang X, Wang Y, Lei H, Su H, Zeng J, Pei Z, Huang R (2015). Inhibition of immunoproteasome reduces infarction volume and attenuates inflammatory reaction in a rat model of ischemic stroke. Cell Death Dis.

[4] Basler M, Mundt S, Muchamuel T, Moll C, Jiang J, Groettrup M, Kirk CJ (2014). Inhibition of the immunoproteasome ameliorates experimental autoimmune encephalomyelitis. EMBO Mol Med.

[5] Muchamuel T, Basler M, Aujay MA, Suzuki E, Kalim KW, Lauer C, Sylvain C, Ring ER, Shields J, Jiang J (2009). A selective inhibitor of the immunoproteasome subunit LMP7 blocks cytokine production and attenuates progression of experimental arthritis. Nat Med.

[6] Moallemian R, Rehman AU, Zhao N, Wang H, Chen H, Lin G, Ma X, Yu J (2020). Immunoproteasome inhibitor DPLG3 attenuates experimental colitis by restraining NF-κB activation. Biochem Pharmacol.

[7] Schmidt C, Berger T, Groettrup M, Basler M (2018). Immunoproteasome Inhibition Impairs T and B Cell Activation by Restraining ERK Signaling and Proteostasis. Front Immunol.

[8] Ding Q, Martin S, Dimayuga E, Bruce-Keller AJ, Keller JN (2006). LMP2 knock-out mice have reduced proteasome activities and increased levels of oxidatively damaged proteins. Antioxid Redox Signal.

[9] Mishto M, Bellavista E, Santoro A, Stolzing A, Ligorio C, Nacmias B, Spazzafumo L, Chiappelli M, Licastro F, Sorbi S (2006). Immunoproteasome and LMP2 polymorphism in aged and Alzheimer's disease brains. Neurobiol Aging.

[10] Bhattarai D, Lee MJ, Baek A, Yeo IJ, Miller Z, Baek YM, Lee S, Kim D, Hong JT, Kim KB (2020). LMP2 inhibitors as a potential treatment for Alzheimer’s Disease. J Med Chem.

[11] Orre M, Kamphuis W, Dooves S, Kooijman L, Chan ET, Kirk CJ, Dimayuga SV, Koot S, Mamber C, Jansen AH (2013). Reactive glia show increased immunoproteasome activity in Alzheimer's disease. Brain.

[12] Gorny X, Saring P, Bergado AJ, Kahl E, Kolodziejczyk MH, Cammann C, Wernecke K, Mayer D, Landgraf P, Seifert U (2019). Deficiency of the immunoproteasome subunit beta5i/LMP7 supports the anxiogenic effects of mild stress and facilitates cued fear memory in mice. Brain Behav Immun.

[13] Chen XY, Fu M, Wan SF, Zhang X, Wang YZ (2021). Association between plasma immunoproteasome and 90-day prognosis after first-ever ischemic stroke. Neural Regen Res.

[14] Chen X, Wang Y, Fu M, Lei H, Cheng Q, Zhang X (2017). Plasma immunoproteasome predicts early hemorrhagic transformation in acute ischemic stroke patients. J Stroke Cerebrovasc Dis.

[15] Mishto M, Bellavista E, Ligorio C, Textoris-Taube K, Santoro A, Giordano M, D'Alfonso S, Listi F, Nacmias B, Cellini E (2010). Immunoproteasome LMP2 60HH variant alters MBP epitope generation and reduces the risk to develop multiple sclerosis in Italian female population. PLoS ONE.

[16] Barnhart CD, Yang D, Lein PJ (2015). Using the morris water maze to assess spatial learning and memory in weanling mice. PLoS ONE.

[17] Zhu NW, Yin XL, Lin R, Fan XL, Chen SJ, Zhu YM, Zhao XZ (2020). Possible mechanisms of lycopene amelioration of learning and memory impairment in rats with vascular dementia. Neural Regen Res.

[18] Knight P, Chellian R, Wilson R, Behnood-Rod A, Panunzio S, Bruijnzeel AW (2021). Sex differences in the elevated plus-maze test and large open field test in adult Wistar rats. Pharmacol Biochem Behav.

[19] Bruijnzeel AW, Knight P, Panunzio S, Xue S, Bruner MM, Wall SC, Pompilus M, Febo M, Setlow B (2019). Effects in rats of adolescent exposure to cannabis smoke or THC on emotional behavior and cognitive function in adulthood. Psychopharmacology.

[20] Chen X, Yao N, Lin Z, Wang Y (2021). Inhibition of the immunoproteasome subunit LMP7 ameliorates cerebral white matter demyelination possibly via TGFbeta/Smad signaling. Evid Based Complement Alternat Med.

[21] Shibata M, Ohtani R, Ihara M, Tomimoto H (2004). White matter lesions and glial activation in a novel mouse model of chronic cerebral hypoperfusion. Stroke.

[22] Chen XY, Wan SF, Yao NN, Lin ZJ, Mao YG, Yu XH, Wang YZ (2021). Inhibition of the immunoproteasome LMP2 ameliorates ischemia/hypoxia-induced blood-brain barrier injury through the Wnt/beta-catenin signalling pathway. Mil Med Res.

[23] Olivera GC, Ren X, Vodnala SK, Lu J, Coppo L, Leepiyasakulchai C, Holmgren A, Kristensson K, Rottenberg ME (2016). Nitric oxide protects against infection-induced neuroinflammation by preserving the stability of the blood-brain barrier. PLoS Pathog.

[24] Ben-Zvi A, Lacoste B, Kur E, Andreone BJ, Mayshar Y, Yan H, Gu C (2014). Mfsd2a is critical for the formation and function of the blood-brain barrier. Nature.

[25] Chen X, Chen L, Lin G, Wang Z, Kodali MC, Li M, Chen H, Lebovitz SG, Ortyl TC, Li L (2022). White matter damage as a consequence of vascular dysfunction in a spontaneous mouse model of chronic mild chronic hypoperfusion with eNOS deficiency. Mol Psychiatry..

[26] Montagne A, Nation DA, Sagare AP, Barisano G, Sweeney MD, Chakhoyan A, Pachicano M, Joe E, Nelson AR, D’Orazio LM (2020). APOE4 leads to blood–brain barrier dysfunction predicting cognitive decline. Nature.

[27] Chen X, Zhang X, Chen T, Jiang X, Wang X, Lei H, Wang Y (2018). Inhibition of immunoproteasome promotes angiogenesis via enhancing hypoxia-inducible factor-1alpha abundance in rats following focal cerebral ischaemia. Brain Behav Immun.

[28] Cai Z, Qiao PF, Wan CQ, Cai M, Zhou NK, Li Q (2018). Role of blood-brain barrier in Alzheimer's Disease. J Alzheimers Dis.

[29] Wagner LK, Gilling KE, Schormann E, Kloetzel PM, Heppner FL, Krüger E, Prokop S (2017). Immunoproteasome deficiency alters microglial cytokine response and improves cognitive deficits in Alzheimer’s disease-like APPPS1 mice. Acta Neuropathol Commun.

[30] Pickering AM, Koop AL, Teoh CY, Ermak G, Grune T, Davies KJ (2010). The immunoproteasome, the 20S proteasome and the PA28alphabeta proteasome regulator are oxidative-stress-adaptive proteolytic complexes. Biochem J.

[31] Groettrup M, Standera S, Stohwasser R, Kloetzel PM (1997). The subunits MECL-1 and LMP2 are mutually required for incorporation into the 20S proteasome. Proc Natl Acad Sci USA.

[32] Kremer M, Henn A, Kolb C, Basler M, Moebius J, Guillaume B, Leist M, Van den Eynde BJ, Groettrup M (2010). Reduced immunoproteasome formation and accumulation of immunoproteasomal precursors in the brains of lymphocytic choriomeningitis virus-infected mice. J Immunol.

[33] Diaz-Hernandez M, Hernandez F, Martin-Aparicio E, Gomez-Ramos P, Moran MA, Castano JG, Ferrer I, Avila J, Lucas JJ (2003). Neuronal induction of the immunoproteasome in Huntington's disease. J Neurosci.

[34] Maltsev A, Funikov S, Burov A, Spasskaya D, Ignatyuk V, Astakhova T, Lyupina Y, Deikin A, Tutyaeva V, Bal N (2021). Immunoproteasome Inhibitor ONX-0914 affects long-term potentiation in murine hippocampus. J Neuroimmune Pharmacol.

[35] Martin SJ, Morris RG (2002). New life in an old idea: the synaptic plasticity and memory hypothesis revisited. Hippocampus.

[36] Biessels GJ, Whitmer RA (2020). Cognitive dysfunction in diabetes: how to implement emerging guidelines. Diabetologia.

[37] Ishii M, Iadecola C (2020). Risk factor for Alzheimer’s disease breaks the blood–brain barrier. Nature.

[38] Islam F, Islam MM, Khan MA, Nafady MH, Islam MR, Akter A, Mitra S, Alhumaydhi FA, Emran TB, Khusro A (2022). Multifaceted role of polyphenols in the treatment and management of neurodegenerative diseases. Chemosphere.

[39] Chodari L, Dilsiz Aytemir M, Vahedi P, Alipour M, Vahed SZ, Khatibi SMH, Ahmadian E, Ardalan M, Eftekhari A (2021). Targeting mitochondrial biogenesis with polyphenol compounds. Oxid Med Cell Longev.

[40] Chen JF, Liu K, Hu B, Li RR, Xin W, Chen H, Wang F, Chen L, Li RX, Ren SY (2021). Enhancing myelin renewal reverses cognitive dysfunction in a murine model of Alzheimer's disease. Neuron.

[41] Bowman GL, Dayon L, Kirkland R, Wojcik J, Peyratout G, Severin IC, Henry H, Oikonomidi A, Migliavacca E, Bacher M, Popp J (2018). Blood-brain barrier breakdown, neuroinflammation, and cognitive decline in older adults. Alzheimer's Dementia.

[42] Singh V, Ubaid S (2020). Role of silent information regulator 1 (SIRT1) in regulating oxidative stress and inflammation. Inflammation.

[43] Yu J, Wang W, Matei N, Li X, Pang J, Mo J, Chen S, Tang J, Yan M, Zhang JH (2020). Ezetimibe attenuates oxidative stress and neuroinflammation via the AMPK/Nrf2/TXNIP pathway after MCAO in rats. Oxid Med Cell Longev.

[44] Hussong SA, Kapphahn RJ, Phillips SL, Maldonado M, Ferrington DA (2010). Immunoproteasome deficiency alters retinal proteasome's response to stress. J Neurochem..

[45] An L, Shen Y, Chopp M, Zacharek A, Venkat P, Chen Z, Li W, Qian Y, Landschoot-Ward J, Chen J (2021). Deficiency of endothelial nitric oxide synthase (eNOS) exacerbates brain damage and cognitive deficit in a mouse model of vascular dementia. Aging Dis.

[46] Zhang J, Yu C, Zhang X, Chen H, Dong J, Lu W, Song Z, Zhou W (2018). Porphyromonas gingivalis lipopolysaccharide induces cognitive dysfunction, mediated by neuronal inflammation via activation of the TLR4 signaling pathway in C57BL/6 mice. J Neuroinflammat.

[47] McMurran CE, Jones CA, Fitzgerald DC, Franklin RJ (2016). CNS remyelination and the innate immune system. Front Cell Dev Biol.

[48] Xiang C, Cong S, Liang B, Cong S (2020). Bioinformatic gene analysis for potential therapeutic targets of Huntington's disease in pre-symptomatic and symptomatic stage. J Transl Med.

